# Emotional Competence Development in Graduate Education: The Differentiated Impact of a Self-Leadership Program Depending on Personality Traits

**DOI:** 10.3389/fpsyg.2021.666455

**Published:** 2021-05-21

**Authors:** Adolfo Montalvo-Garcia, Margarita Martí-Ripoll, Josep Gallifa

**Affiliations:** ^1^School of Psychology, Education and Sport Sciences (FPCEE Blanquerna), Ramon Llull University, Barcelona, Spain; ^2^ESADE Business School, Ramon Llull University, Barcelona, Spain

**Keywords:** emotional competence development, self-leadership, personal growth, graduate education, personality traits, big five

## Abstract

There is little research on the effectiveness of self-leadership programs (SLPs) in graduate education based on the progress in emotional competences development (ECD), and only a few of the studies incorporate its relationship with personality traits (PTs). This article studies the differentiated impact of an optional SLP, which has eight workshops with a learner-centered and experiential approach, depending on PTs. With a quasi-experimental *ex post facto* design, students' scores in EDC were analyzed according to their PT extremes: *introversion, antagonism, lack of direction, neuroticism*, and *closed to experience*. ANCOVA tests, with ECD pretest as a co-variable, were applied for each PT. The results indicated that the SLP presented a differentiated impact in ECD in four of the five PTs: neuroticism, introversion, antagonism, and lack of direction. These findings can be a key element for the participating students in SLPs because self-leadership requires self-knowledge. ECD can contribute to more integral learning in the graduate education experience, enhancing the preparation for the world of work.

## Introduction

### Self-Leadership and ECD

Emotional competences development (ECD) is an essential part of the integral development of people and can project their possible benefits in the academic, social, family, and work spheres (Lechner et al., 2019). The United Nations (UN), United Nations Educational, Scientific and Cultural Organization (UNESCO), and the Organization for Economic Co-operation and Development (OECD) agree to confer to education the purpose of training people in a holistic way in order to develop the necessary capacities and abilities to respond and solve the difficulties of life (Zabala and Arnau, 2008). Nevertheless, the presence of emotional competences (ECs) in the curricula of the European Higher Education Area (EHEA) is, in general, scarce compared to the technical and instrumental ones, although there are *a priori* associated benefits of these competences for the world of work and to have a more balanced and full life. These aspects have been widely reported in the literature. In effect, from the point of view of the students and their well-being, emotions are playing a fundamental role in the teaching–learning processes and it is essential to be experiencing more comprehensive training (Zabala and Arnau, 2008; Pekrun and Perry, 2014; Amutio et al., 2015). From the pedagogical perspective, this part of the development of students is carried out in the EHEA through the also denominated as generic competences, although it remains a field with many gaps that require further exploration and solutions (Gallifa and Garriga, 2010; Mourshed et al., 2014; ILO, 2015). Finally, from the point of view of work, self-leadership programs (SLPs), and the development of associated ECs contribute to a better professionalization of students (Klaus, 2010; Rath and Harter, 2010) in a scenario where the OECD declares that ECs are an essential aspect in the future of the work, demanding for a good initial EC education, which serves as a basis for further ECD in their professional careers (OECD, 2019).

ECD can be a way to operationalize *self-leadership* progress (Goleman, 2017). The only longitudinal research on the assessment of a leadership program, the Leadership Development Programme (LEAD), with socio ECs in higher education, has been developed by Boyatzis and Cavanagh (2018). It revealed mixed results, with positive effects on adaptability, inspiring leadership, and influence and, on the contrary, with no effects in conflict management, coaching, orientation to achievement, and teamwork.

On the other hand, the relationship between emotional intelligence (EI) and personality traits (PTs) has been extensively studied, and for many authors, EI and PTs are two overlapping constructs (Van Rooy and Viswesvaran, 2004; Van der Linden et al., 2017; Alegre et al., 2019). The EI construct, considered as a trait related to PTs, was developed (Petrides and Furnham, 2001). In that perspective, the PTs—specifically measured with the Big Five instrument—provide information about the usual responses and reactions offered to external stimuli as part of EI (Bisquerra et al., 2015; Navarro, 2017). ECs and EI are two constructs so close that, in many studies, they are repeatedly interchanged (Fragoso-Luzuriaga, 2015). To differentiate between them, ECs can be understood as more flexible and finalist, and it can also be used to answer the question of what can be done with emotional awareness (Boyatzis et al., 2015).

An EC approach in graduate education allows working from this logic, complementing the more regular work with the specific or technical competencies. From the point of view of a graduate student who follows an SLP, knowing one's PTs is vital, which is a piece of essential information because the development of self-leadership has its foundation, in all EC models, in self-knowledge (Montalvo, 2020). Because the PTs are a constitutive element of the ECs, along with “motives, abilities, and other aspects of the Self” (Boyatzis, 1982, p. 21), then it would be interesting to find out what are the particular PTs of graduate students who can benefit more from participating in an SLP in order to develop their self-leadership competence.

In this regard, besides PTs being stable constructs over time (McCrae, 2018), Damian et al. (2019) reported in a longitudinal 50-year period study that personality is malleable when it is considered from a life span perspective because of the process of development and maturation from adolescence to retirement.

PTs are the unique dispositions of an individual with no clear heritage influence (McCrae, 2018). Heritage is estimated in values near 50% in twins and is lower with other relatives such as uncles or nephews (Jang et al., 1998, cited in McCrae, 2018). PTs are a constitutive element of EC; they are a subset of its totality (Kankara and Suarez-Alvarez, 2019). In this sense, it is interesting that a recent study, not about ECs, found that socio-emotional skills are constituted by “PT, motivation, preferences and values” (Lechner et al., 2019, p. 427). In this study socio-emotional skills predicted adulthood incomes, health or social participation, formal education level, and cognitive skills.

Considering what has been explained so far, it would be interesting to find out what are the particular PTs more benefited from participating in an SLP in graduate students who intended to develop their ECD.

### The Big Five Model of PTs

The Big Five model (BFM) of PTs is a development that explains personality on five factors. This model has been translated into many languages and has been used/replicated profusely. It demonstrated consistency and validity, and in this sense, the sub-dimensions of the model make up the acronym OCEAN, a word that symbolizes the amplitude and applicability of the model. The BFM is the most recognized and used today's model to address the question of personality (Rossberger, 2014). There is a danger identified in the literature about the use of the personality traits language because it may have an evident positive or negative bias for the extremes of each trait (Cain, 2013; Noya and Vernon, 2019). This fact makes evident the necessity to take special care and have neutrality when this information is used for describing the traits of a concrete individual. The following lines illustrate some of the main positive aspects of each one of the extremes for each PT in the BFM in a neutral manner.

*Extraversion* is related to assertiveness and sociability and being outgoing (Lechner et al., 2019), as opposed to the immobility and reserved character with which *introversion* is described (Perkins et al., 2010). According to the extraverted ideal, the space where leadership takes place is in being with other people (Cain, 2013). In this aspect, extraversion appears to be a desirable trait in leadership: both to enjoy social relationships as well as being a *sine qua non* condition to be able to emotionally resonate with people (Goleman et al., 2004). From a holistic approach, however, it is not possible to understand the leadership phenomena without considering, as does the emotional labor theory (Grandey, 2000), all the factors involved, not only the individual but also the organizational. From this complexity, introverts present some traits that give them an advantage in organizations: they are less dominant and use, accordingly, less authoritarian language when they are occupying formal leadership positions (Tost et al., 2013). This means that they usually can be developing more respect toward their teams and also enable other people to lead (Grant et al., 2011; Hunter et al., 2013), which implies a leader-of-leaders approach. The literature has also identified that the combination of introverted leaders with proactive followers is more effective (Grant et al., 2011), finding a broader compatibility of introversion with the *servant leader* style (Hunter et al., 2013).

The facets that make up the *openness to experience* PT are the approach to fantasy, aesthetics, feelings, action, ideas, and values (Costa and McCrae, 1992). Openness to experience is manifested when there is a need to try new options and to be open to unconventional ideas or new beliefs, which are challenging the basic assumptions of individuals (Perkins et al., 2010). Openness has to do with a predisposition to say yes and to take advantage of the windows of opportunity (Kotter, 2012), both professional and vital that they can be opened for each person. From the antagonistic perspective, at the other extreme of the PT—*closed to experience*—it has to be noted that leadership also has to do with knowing to *say no*, for example when it is pertinent to be marking one's profile, to defend the achievement of personal or organizational visions, for instance, or also when the person in front is fully oriented to power motivations (McClelland and Burnham, 2003).

*Neuroticism* is the only trait in the BFM that is inverted or with negative characteristics as opposed to balance: Amirazodi and Amirazodi (2011) found that this trait manifests itself in a greater predisposition to stress, easiness of frustration, and insecurity in relationships in university students. Kumari and Sharma (2016) established that neuroticism affects subjective well-being, while Teng and Liu (2013) associated *neuroticism* with propensity for depression. For Sushma and Batra (2015), neuroticism is a trait of conformist and disorganized people, individuals who do not pay attention, and those who live their life in a reserved way. This extreme is associated with traits such as anxiety, hostility, impulse, or depression (McCrae and Terracciano, 2005), propensity to have bad feelings (McCrae and Terracciano, 2005; Rossberger, 2014), lack of self-control (Mao et al., 2018), and lack of self-esteem (Kaur and Singh, 2019). Low levels of neuroticism mean, for university students, an improvement in emotional stability (Hsieh et al., 2011). Anyhow, there is also a positive implication of high neuroticism, and this has to do with perfectionism (Stricker et al., 2019) since it requires the tension and continuous action typical of this trait, which is an essential requirement to achieve high-quality results.

*Agreeableness* is the degree of friendship and cooperation offered to show pro-social behaviors (Perkins et al., 2010). Moreover, according to Costa and McCrae (1992), the agreeable extreme of this PT has to do with altruistic and sensitive people, specifically in the following facets: trust, frankness, altruism, complacency, modesty, and tenderness. Individuals at the agreeable extreme are open to the needs of others (Tov et al., 2016). This trait is manifested in people who cooperate with others (Lechner et al., 2019), adopt more constructive positions in conflict resolution, are more oriented to cooperation in teamwork, and spend more time suppressing negative emotional states inside groups (Tobin et al., 2000; Jensen-Campbell and Graziano, 2001). But, although people who score low in agreeableness appear as manipulative, selfish, and can be rude (Digman, 1990), in some situations, either due to the urgency of a response or due to the competencies and commitment of the teams (Hersey and Blanchard, 1976), the exercise of effective leadership implies the adoption of authoritarian styles, which nevertheless will produce dissonant effects on people (Goleman et al., 2004).

The facets of *consciousness*, at the conscious extreme of this PT, are competence, order, obedience, achievement orientation, self-discipline, and deliberation (Costa and McCrae, 1992). The conscientiousness trait provides an in-depth understanding of both the constituent elements of social phenomena as well as their interrelations. Consciousness improves achievement, organization, efficiency, care, and interdependence (Goldberg, 2001), as well as the internalization of social norms (De Raad and Schouwenburg, 1996). It has to do with realizing the consequences of one's behaviors and therefore is linked to the responsibility toward the related tasks assigned to the individual (Perkins et al., 2010). This trait is close to self-discipline, self-control, and persistence (Muthu et al., 2010), and for some authors, it is the PT that best explains the academic performance in university (De Raad and Schouwenburg, 1996; Trapmann et al., 2007). However, the unconscious pole of this PT is also critical in leadership, understood as the start of the processes of change toward creations, innovations, or even transgressions of the established. It is one of the first stages in the purpose of a leader, who is someone who questions the established order of things and who goes ahead to convince everyone else to turn his idea into a reality.

Regarding ECs, Bisquerra (2003) conceived emotional education as “an educational process, continuous and permanent, which aims to promote the development of emotional competencies as an essential element of the integral development of the individual, to prepare them for life. All of this is aimed at increasing personal and social well-being” (p. 27), EC being “the set of knowledge, capacities, abilities, and attitudes necessary to understand, express and regulate emotional phenomena appropriately” (p. 24).

The constituent elements of the EC model proposed by Bisquerra and Pérez-Escoda (2007) are specified in five blocks of competences, known as socio-personal, as follows: emotional consciousness, emotional regulation, emotional autonomy, social competences, and emotional well-being.

### The Self-Leadership Program

The studied SLP is a voluntary and transversal program in graduate studies open to all students at the master's level of a business school in the city of Barcelona. The program aims to complement the specific training received in the regular subjects of each degree, starting with becoming aware and strategically mobilizing EC, fostering a space for combining reason and emotion (Alburquerque et al., 2018) for self-leadership.

The aims of this program are as follows:

- Develop the personal leadership abilities of participants as a base for good teamwork.
- Enhance the skills and key competencies to ensure the effective interrelation of people within work teams in order to improve their contributions.
- Work to achieve that each person understands and becomes aware of their individual and collective responsibility.
- Understand that the ability to adapt to new environments is not a reaction but a systematic set of proactive actions.

The SLP is composed of eight workshops, for 3 h each, with a highly experiential approach (Kolb, 1984) that consists of applying the logic of the self-directed learning theory (Kolb and Boyatzis, 1970; Canboy et al., 2016). Considering the simultaneous work with the triune brain (MacLean, 1949), this experiential approach proposes a lot of activity on the part of each student, with the idea of going beyond the transmission of knowledge that activates the neocortex, also reaching through these experiences in the activation of the emotional or limbic brain.

The architecture of the program is based on the socio-emotional competences model (Boyatzis et al., 2000), which establishes that self-leadership begins with self-knowledge. For this to facilitate the first step, the BFM of PT is incorporated into the SLP. In this way, each student can grow personally in terms of ECD from the metacognitive knowledge of his/her specific individual personality traits. The ECD process continues with how individuals are entering into relationships with other people to constitute groups. Advancing in the model implies going beyond the ego and unfolding the “we,” where each person develops a kind of leadership role in social situations. Finally, students work in situations implied in the leadership roles and what kind of influence deploys each self in them.

The SLP program is delivered by three coordinated professors.

### Hypothesis

Our experience made us think that introverted students are taking more advantage of the SLP because, through their participation, we observed that they gained emotional consciousness, confidence, and activated and developed the rest of their EC. Students usually reported these changes. Because EC is constituted, in addition to other elements, by PTs and skills (Boyatzis, 1982), our purpose was to find out the growth in the ECD measures for those introverted, who present a PTs that are far from the “extrovert ideal” (Cain, 2013), after participating in the self-leadership program. We hypothesized similar findings with the other PTs. Regarding the extreme *closed to experience*, we considered that leading means manifesting new ideas into reality from innovative approaches, which means placing oneself in an attitude of permanent challenge (Perkins et al., 2010). Once again, the logic is that the group of students who are closed to experience is expected to present more potentiality in the developmental path as they are away from this approach, from the ideal open characteristics of this PT. Similarly, moving to *neuroticism*, some of the traits of people closest to neuroticism, such as stress, frustration, or insecurity (Amirazodi and Amirazodi, 2011), made us think that maybe these are the people who most need to reflect upon and rethink their approaches. Once again, the subgroup of students with the highest neuroticism is the one that seems to have more differential in ECD. In terms of the *antagonist* trait, similarly, participants are those who are separated from collaboration or friendship (Perkins et al., 2010), which guides the quality of teamwork. Also, they are expected to have room for improvement. Finally, for students with *lack of direction* traits, because consciousness is a construct that explains part of the academic performance at the university level (Trapmann et al., 2007), as well as success in the world of work (Barrick et al., 2001), people with the highest values in lack of direction are expected to take more advantage of the SLP, gaining a deeper understanding of the environment, of the relationships established, and of the consequences of both the different approaches to reality and of their decisions.

Therefore, the hypothesis formulated in this research was the following: There are differentiated effects of the self-leadership program in emotional competences development according to the personality trait extremes: *introversion, lack of direction, neuroticism, closed to experience*, and *antagonism*.

## Materials and Methods

### Participants

The sample comprised students enrolled in different master's degrees in management of a business school located in Barcelona. This student population is international, similar to other master's degree populations in other business schools. In this case, students came from Spain, Peru, Colombia, Mexico, Guatemala, Ecuador, Venezuela, Italy, Argentina, Chile, Panama, Bolivia, Costa Rica, El Salvador, and Uruguay.

The total population in the school was 270 students. Sixty-three of them enrolled in the SLP. This sample formed the experimental group. Sixty-three others were randomly selected from the remaining students. The number of student participants in this study was *n* = 126 (62.5% females, *m*_age_ = 31.6 years). Students were assigned to the experimental condition according to the natural groups design, which means that the group is composed of students who freely decided to join the SLP. Students in the control group were part of the same international population. Their initial PTs, ECs, or even cultural backgrounds were equally distributed in both experimental and control groups.

### Instruments

Two instruments were selected for this study. Both had been psychometrically validated and had been widely used in research.

The EDQ-A35, the Emotional Development Questionnaire in Adults, the second version with 35 items, measures self-perception in this construct. It is an instrument with a self-information modality, where eight items collect information on emotional consciousness, eight items on emotional regulation, five items on social competences, six items on emotional autonomy, and, lastly, eight items on life and well-being. The Cronbach's α of this instrument is 0.92. It contains reverse items that place participants in negative introspections or scenarios, where a response of the highest “agree” range implies little development of the explored dimension.

The Spanish version of the Big Five Inventory (BFI) (Rodríguez-Fornells et al., 2001) measures personality traits. It is a 44-item instrument with an internal consistency of a Cronbach's α of 0.95. Its Likert scale ranges from “1: Disagree strongly” to “5: Agree strongly.” Extraversion has eight items, agreeableness has nine items; conscientiousness nine items; neuroticism eight items, and openness has 10 items. Like the EDQ-A35, it incorporates reverse items and is self-reported.

### Design

We applied a quasi-experimental design *ex post facto*. Students were assigned according to the natural groups design, which means groups are composed of students who freely decided to join the SLP (experimental group) and those who did not participate (control group). Students were part of the same population of students and with equivalent traits. To control the initial situation, we decided to pass the EDQ-A35 as the pretest. This allowed having a variable with the initial scores. EDQ-A35 posttest was the dependent variable.

*Ex post facto*, we applied an analysis of covariance (ANCOVA) for each personality trait, PT being the independent variable and the EDQ-A35 pretest the covariable. To constitute the groups for the five ANCOVAs, we applied the following procedure: For each PT, we calculated the mean values of all measures, independently of whether they participated or not in the SLP. These means were considered the cutting points, and subjects with lower scores than these mean values from the experimental and control groups form two subgroups, respectively. For instance, the mean for introversion was 3.51, so subgroup 1.1 was created with *n* = 30 students, while subgroup 2.1 had 37 students. These subgroups were the ones used in the analysis of introversion.

This operation was repeated for each PT, forming successive subgroups and respective ANCOVAs. Regarding the trait closed to experience, the average was 3.83, and so experimental subgroup 1.2 had 29 students and the control subgroup 2.2 had 32 students. Moving to neuroticism, the average of this reverse PT was 2.67, so the experimental subgroup 1.3 had 35 students and the control subgroup 2.3 had 28. For the antagonism trait, the average value was 3.69: the experimental subgroup 1.4 had 32 students and, on the other hand, the control subgroup 2.4 had 34 students. Finally, the average for students with a lack of direction was 3.81: the experimental subgroup 1.5 had 27 students and subgroup 2.5 had 44.

The analysis in each ANCOVA of EDQ-A35 with EDQ-A35 pretest as a co-variable allows analyzing the gains with participation in the SLP because of the possibility of extraction in the dependent variable of the regression of the initial scores. These gains will be tested in each PT subgroup. Statistically significant differences suppose effects of the personality trait in the gains for participating in the SLP.

### Process

Just before starting the SLP, the EDQ-A35 and BFI questionnaires were administered by three professors. The participants were given an explanation about the study and were asked whether they voluntarily agree to participate. In return, they would be offered an individualized report of their evolution in the ECs, for all those who will participate in the entire program and contribute their data to this work. The time required to complete the questionnaires was approximately 40 min. Before starting to answer the questions, the instructions to fill out the questionnaires were reviewed, and it was explained to students that there are no good or bad answers, that this work would only make sense if the answers are honest.

Just after the end of the last workshop, the whole class groups were asked to respond to the EDQ-A35 and BFI questionnaires once again, thus completing the post-data collection within the framework of the quasi-experimental design.

According to the terms of use of the EDQ-A35, the results were sent to its author, who returned the obtained scores according to their theoretical model (Bisquerra and Pérez-Escoda, 2007). For the evaluation of the personality traits, the scores were obtained by following the instructions of the Big Five Inventory (John and Srivastava, 1999).

### Analysis of Data

SPSS 26 software was used for the statistical analysis. The significance standard of *p* = 0.05 was adopted. After reviewing the assumptions of normality with the Kolmogorov–Smirnov test and the homogeneity of variances with the Levene test, the ANCOVA technique was applied, as has been explained. The ANCOVA test allows the analysis of variance, a statistical technique that makes it possible to determine differences in the effects of an independent variable between two or more groups based on quantitative answers, in our case in the EDQ-A35, incorporating the regression of the pretest results, that is, the EDQ-A35 score that each student already had before the SLP.

In the case of not fulfilling the normality and homogeneity requirements, the non-parametric Kruskal–Wallis test was used to obtain a complementing perspective of the group comparisons, in this case, without considering pretest as a covariable. Both ANCOVA and the Kruskal–Wallis test are inferential; that, is they allow the extrapolation of the results from the sample to the entire population.

### Ethical Approach

This investigation is focused on the attempt to contribute to the training of university students. They enrolled in an SLP with the idea of complementing and balancing their competence development, not only from the technical side but also from the ECD. The purpose of SLP is to deal with an initial lack of self-awareness as a starting point, and with a progressive activation of their EC and, therefore, their self-leadership competences. From an operational point of view, informed consent had been collected from the institution where the empirical data were gathered and from the participating students when it was necessary. The confidentiality of personal data has been preserved. All along the SLP, the students were encouraged to express themselves freely for their self-leadership development.

## Results

### Normality and Homogeneity of the Distributions

Normality and homogeneity had been verified. In subgroups 1.5 and 2.5 of lack of direction, the distribution did not fulfill the normality assumption; therefore, the equivalent non-parametric Kruskal–Wallis test was applied to complement the results in that PT. Another requirement is that the covariable needs to be independent from the independent variable. It is the case of the covariable pretest and PTs.

### Descriptive Statistics

**Figure 1:**
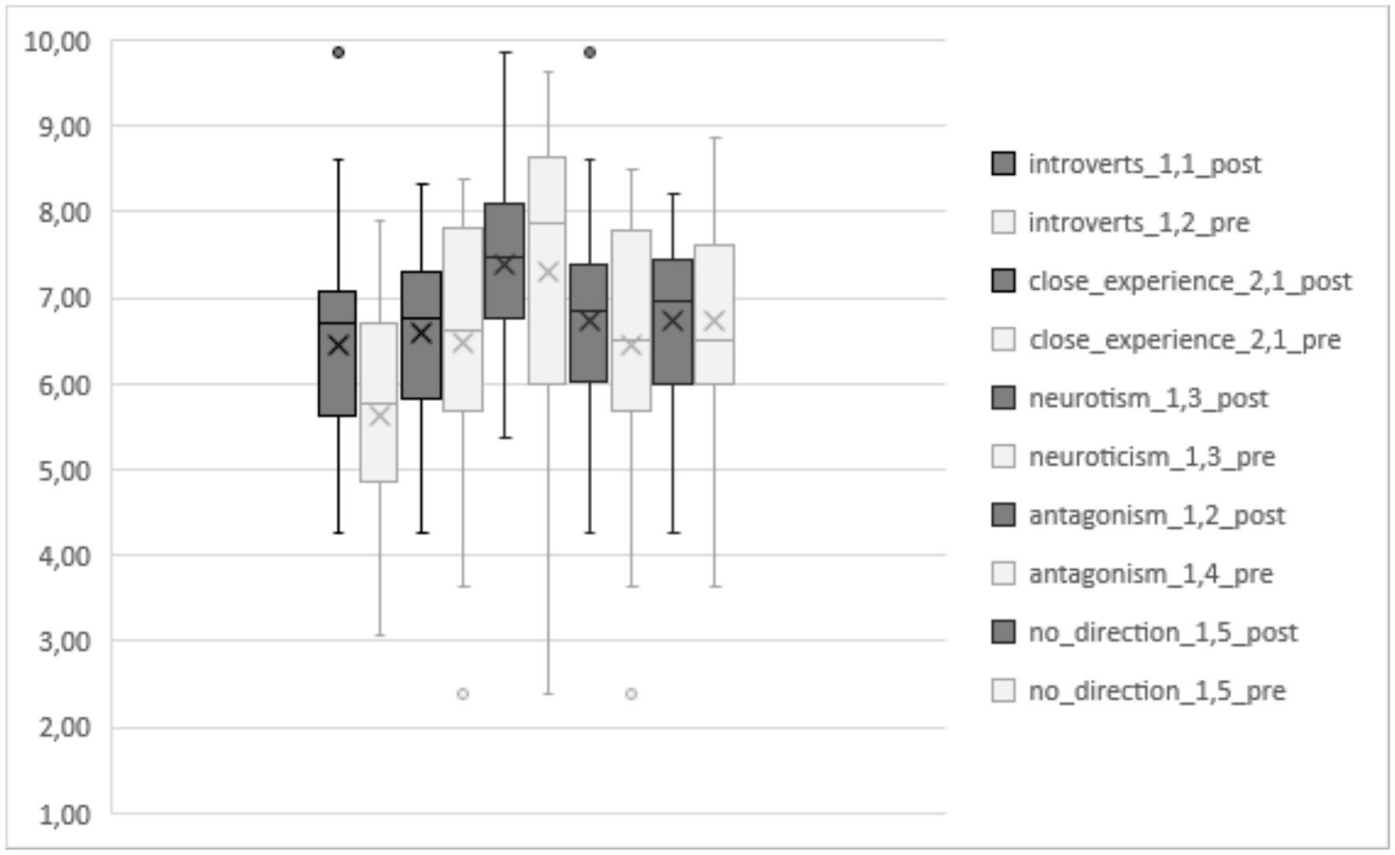
Emotional competence (EC) evolution pretest–posttest for the five personality traits (PTs) in the experimental group.

Figure 1 shows the evolution pretest–posttest for each of the PTs in the experimental group. In all the PTs analyzed, the averages scored higher in the post-test, except the neuroticism PT where the pretest scored higher than the posttest.

Regarding the post-test situation, Table 1 offers the group composition, the mean, and the standard deviation values obtained in the EC total in the post-test for both subgroups 1 and 2. The means always appear lower in every single PT in subgroup 2 compared with subgroup 1.

**Table 1:** Emotional competences development (ECD) posttest in experimental and control subgroups.

**Post stage**	**Experimental group 1**			**Control group 2**		
	***N***	**Mean**	**SD**	***N***	**Mean**	**SD**
Extroversion (subgroups 1.1 and 2.1)	30	6.44	1.21	37	5.83	1.23
Openness to experience (subgroups 1.2 and 2.2)	29	6.53	0.92	32	6.25	1.09
Neuroticism (subgroups 1.3 and 2.3)	35	6.48	1.07	28	5.81	1.37
Agreeableness (subgroups 1.4 and 2.4)	32	6.96	1.05	34	5.85	1.28
Consciousness (subgroups 1.5 and 2.5)	27	6.79	0.81	44	6.53	0.98

**Figure 2:**
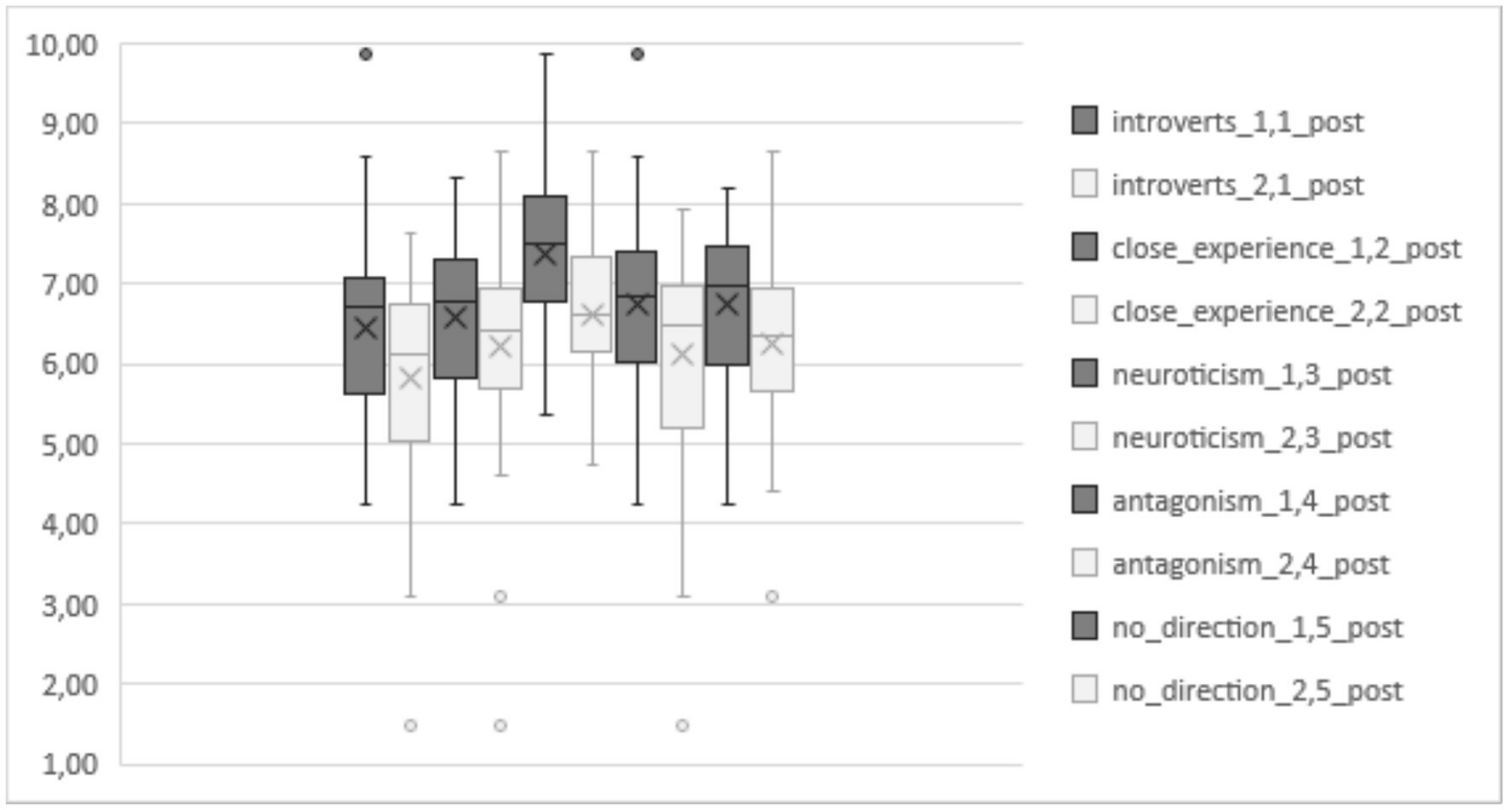
Emotional competence (EC) posttest means in subgroups 1 and 2 (experimental and control subgroups).

The post-test situation is represented in Figure 2. As is visible, different means are shown for each selected subgroup of the PTs, conforming a quite regular pattern, always with higher mean marks for subgroup 1.

### ANCOVA Results

The results of the ANCOVA test for introversion [*F*_(1,66)_ = 7.194, *p* = 0.009, η^2^ = 0.101] can be found in Table 2. The significance level is lower than *p* < 0.05; therefore, there are differences between subgroups 1.1 and 2.1.

**Table 2:** ANCOVA results for emotional competences development (ECD): introversion.

**Between-subjects test (dependent variable: EC_total introversion)**						
**Source**	**Type III sum of squares**	***df***	**Mean square**	***F***	**Sig**.	**Partial** ***η***^**2**^
Corrected model	38.559^a^	2	19.279	19.124	0.000	0.374
Intercept	25.183	1	25.183	24.980	0.000	0.281
Pre_EC_total introversion	32.411	1	32.411	32.150	0.000	0.334
Subgroups 1.1 and 2.1	7.252	1	7.252	7.194	0.009	0.101
Error	64.521	64	1.008			
Total	2,597.736	67				
Corrected total	103.079	66				

a *R^2^ = 0.374 (adjusted R^2^ = 0.355)*.

The ANCOVA that compared subgroups 1.2 and 2.2, in Table 3 offers the following results: *F*_(1,28)_ = 2.398, *p* = 0.127, η^2^ = 0.000. Therefore, the PT closed to experience does not present significant differences in taking advantage of the SLP.

**Table 3:** ANCOVA results for emotional competences development (ECD): close to experience.

**Between-subjects test (dependent variable: EC_total closed to experience)**						
**Source**	**Type III sum of squares**	***df***	**Mean square**	***F***	**Sig**.	**Partial** ***η***^**2**^
Corrected model	11.307^a^	1	11.307	15.902	0.000	0.371
Intercept	17.562	1	17.562	24.700	0.000	0.478
Pre_EC_total introversion	11.307	1	11.307	15.902	0.000	0.371
Subgroups 1.2 and 2.2	0.000	0	2.038	2.398	0.127	0.000
Error	19.197	27	0.711			
Total	1,290.315	29				
Corrected total	30.504	28				

a *R^2^ = 0.371 (adjusted R^2^ = 0.347)*.

Regarding neuroticism, in Table 4, the results of the ANCOVA test are: *F*_(1,62)_ = 13.292, *p* = 0.001, η^2^ = 0.181. For this PT, significant differences between subgroups 1.3, and 2.3 are found because the condition of *p* < 0.01 is accomplished. The PT neuroticism affects the ECD.

**Table 4:** ANCOVA results for emotional competences development (ECD): neuroticism.

**Between-subjects test (dependent variable: EC_total neuroticism)**						
**Source**	**Type III sum of squares**	***df***	**Mean square**	***F***	**Sig**.	**Partial** ***η***^**2**^
Corrected model	31.358^a^	2	15.679	27.204	0.000	0.476
Intercept	30.038	1	30.038	52.118	0.000	0.465
Pre_EC_total introversion	22.251	1	22.251	38.607	0.000	0.392
Subgroups 1.3 and 2.3	7.661	1	7.661	13.292	0.001	0.181
Error	34.581	60	0.576			
Total	3,185.222	63				
Corrected total	65.938	62				

a *R^2^ = 0.476 (adjusted R^2^ = 0.458)*.

Moving to antagonism, the ANCOVA test in Table 5 reveals: *F*_(1,65)_ = 6.812, *p* = 0.011, η^2^ = 0.098. Again, a level of significance lower than 0.05 shows changes between subgroups 1.4 and 2.4; there is an effect of antagonism in the SLP.

**Table 5:** ANCOVA results for emotional competences development (ECD): antagonism.

**Between-subjects test (dependent variable: EC_total antagonism)**						
**Source**	**Type III sum of squares**	***df***	**Mean square**	***F***	**Sig**.	**Partial** ***η***^**2**^
Corrected model	52.547^a^	2	26.273	27.956	0.000	0.470
Intercept	21.280	1	21.280	22.642	0.000	0.264
Pre_EC_total introversion	45.903	1	45.903	48.842	0.000	0.437
Subgroups 1.4 and 2.4	6.402	1	6.402	6.812	0.011	0.098
Error	59.209	63	0.940			
Total	2,832.423	66				
Corrected total	111.755	65				

a *R^2^ = 0.470 (adjusted R^2^ = 0.453)*.

Finally, in Table 6, the results of the ANCOVA contrasting subgroups 1.5 and 2.5 are summarized as: *F*_(1,70)_ = 6.966, *p* = 0.010, η^2^ = 0.093. The level of significance of 0.01 supports differences between the subgroups, which means that the independent variable lack of direction in students affects the results of the dependent variable SLP. In this *lack of direction* PT, the Kolmogorov–Smirnov test revealed a non-normal distribution for experimental subgroup 1.5, with a significance of 0.002. The Kruskal–Wallis test with no “pretest” co-variable offered a significance below 0.05 (exactly 0.031), which would confirm the differentiation between subgroups 1.5 and 2.5 from the non-parametric statistics.

**Table 6:** ANCOVA results for emotional competences development (ECD): without direction.

**Between-subjects test (dependent variable EC_total without direction)**						
**Source**	**Type III sum of squares**	***df***	**Mean square**	***F***	**Sig**.	**Partial** ***η***^**2**^
Corrected model	39.993^a^	2	19.997	37.324	0.000	0.523
Intercept	24.608	1	24.608	45.931	0.000	0.403
Pre_EC_total introversion	36.345	1	36.345	67.838	0.000	0.499
Subgroups 1.5 and 2.5	3.732	1	3.732	6.966	0.010	0.093
Error	36.432	68	0.536			
Total	3,022.983	71				
Corrected total	76.425	70				

a *R^2^ = 0.523 (adjusted R^2^ = 0.509)*.

### Estimation and Signification of the Effects

The estimated means for each PT in the experimental/control subgroups are shown in Tables 7–11.

**Table 7:** ANCOVA estimated means for introversion.

**Dependent variable: EC post**								
**Subgroups**	**Mean**	**SE**	**95% Confidence interval**		**Bootstrap for mean**^**a**^			
			**Lower bound**	**Upper bound**	**Bias**	**SE**	**95% Confidence interval**	
							**Lower**	**Upper**
Subgroup 1.1	6.47^b^	0.183	6.10	6.83	0.004	0.199	6.10	6.85
Subgroup 2.1	5.80^b^	0.165	5.48	6.13	0.016	0.191	5.41	6.18

a *Unless otherwise noted, Bootstrap results are based on 1,000 bootstrap samples*.

b *Covariates appearing in the model are evaluated at the following value: EC_pre = 5.67*.

**Table 8:** ANCOVA estimated means for closed to experience.

**Dependent variable: EC post**								
**Subgroups**	**Mean**	**SE**	**95% Confidence interval**		**Bootstrap for mean**^**a**^			
			**Lower bound**	**Upper bound**	**Bias**	**SE**	**95% Confidence interval**	
							**Lower**	**Upper**
Subgroup 1.2	6.57^b^	0.171	6.24	6.93	−0.015	0.170	6.23	6.92
Subgroup 2.2	6.22^b^	0.163	5.89	6.54	0.009	0.212	5.79	6.61

a *Unless otherwise noted, Bootstrap results are based on 1,000 bootstrap samples*.

b *Covariates appearing in the model are evaluated at the following value: EC_pre = 5.90*.

**Table 9:** ANCOVA estimated means for neuroticism.

**Dependent variable: EC post**								
**Subgroups**	**Mean**	**SE**	**95% Confidence interval**		**Bootstrap for mean**^**a**^			
			**Lower bound**	**Upper bound**	**Bias**	**SE**	**95% Confidence interval**	
							**Lower**	**Upper**
Subgroup 1.3	7.35^b^	0.128	7.09	7.61	0.008	0.141	7.07	7.63
Subgroup 2.3	6.65^b^	0.144	6.36	6.93	0.000	0.166	6.32	6.98

a *Unless otherwise noted, Bootstrap results are based on 1,000 bootstrap samples*.

b *Covariates appearing in the model are evaluated at the following value: EC_pre = 6.84*.

**Table 10:** ANCOVA estimated means for antagonism.

**Dependent variable: EC post**								
**Subgroups**	**Mean**	**SE**	**95% Confidence interval**		**Bootstrap for mean**^**a**^			
			**Lower bound**	**Upper bound**	**Bias**	**SE**	**95% Confidence interval**	
							**Lower**	**Upper**
Subgroup 1.4	6.74^b^	0.171	6.40	7.08	−0.020	0.192	6.34	7.10
Subgroup 2.4	6.12^b^	0.166	5.79	6.45	−0.010	0.207	5.70	6.48

a *Unless otherwise noted, Bootstrap results are based on 1,000 bootstrap samples*.

b *Covariates appearing in the model are evaluated at the following value: EC_pre = 5.97*.

**Table 11:** ANCOVA estimated means for lack of direction.

**Dependent variable: EC post**								
**Subgroups**	**Mean**	**SE**	**95% Confidence interval**		**Bootstrap for mean**^**a**^			
			**Lower bound**	**Upper bound**	**Bias**	**SE**	**95% Confidence interval**	
							**Lower**	**Upper**
Subgroup 1.5	6.73^b^	0.141	6.45	7.02	−0.001	0.141	6.47	7.02
Subgroup 2.5	6.26^b^	0.110	6.04	6.48	−0.007	0.153	5.94	6.56

a *Unless otherwise noted, Bootstrap results are based on 1,000 bootstrap samples*.

b *Covariates appearing in the model are evaluated at the following value: EC_pre = 6.04*.

The effects of the experimental treatment are as follows: 0.662, *p* < 0.009 for introverted; 0.472, *p* < 0.010 for lack of direction; 0.703, *p* < 0.001 for neurotic; and 0.623, *p* < 0.011 for antagonistic. The experimental condition did not have effects on closed to experience.

## Discussion

Although differences were found between the pre-test and post-test results in the experimental group and also in the post-test between the experimental and control groups with the pre-test results as a co-variable, these were not included in the analysis because of their partial character. The results in terms of general ECD directly caused by participating in an SLP would need further exploration. This not absolute clear result is consistent with that reported in previous research (Boyatzis and Cavanagh, 2018).

The study on the effects of PTs on ECD as a consequence of participating in the SLP revealed interesting patterns. This work reveals that PTs present important and very significant effects on the ECD of graduate students participating in an SLP when a dynamic EC approach is adopted, with an explicit aim of promoting an intentional personal change, as other authors did (Boyatzis and Akrivou, 2006; Boyatzis, 2008, 2014). In this investigation, almost all of the PT extremes contributed to ECD. Four out of five PTs had effects in taking advantage of participating in an SLP in this order: neuroticism, introversion, antagonism, and lack of direction. However, the PT closed to experience had no effects. PTs contribute decisively to the ECD of students in a graduate education SLP; therefore, the inclusion of PTs in an SLP approach allows the possibility of anticipating relevant information about which students may be benefited more from their participation in an SLP by improving differentially their ECD.

The effects found are independent because personality traits are independent traits and independent measures. Nevertheless, these effects only have a predictive characteristic, with the best gain when different traits are implied. Summative effects cannot be assured with the model. Predictive general models would need the consideration of more variables in addition to personality.

The effects are generalizable to a general population, from this sample, because we used inferential statistics. The effect of correcting ECD post-test with the ECD pre-test measure as a co-variable made possible an analysis of the SLP as an effective dependent variable affected by independent PT effects.

The EC construct incorporates PTs as well as, as has been explained, “motives, abilities, and aspects of the self” (Boyatzis, 1982. p. 21). In this sense, the students with personality profiles with traits less desirable than the professional profiles of social sciences, specifically with traits of neuroticism, introversion, antagonism, and lack of direction, have the possibility, through participation in an SLP, to have their ECD improved significantly. In the studied program, this can be achieved with a process of self-knowledge after taking the free personal decision to be fully involved in this process. Taking into consideration that PTs are stable (McCrae, 2018; Damian et al., 2019) in the short frame of an SLP of only one academic year, ECD changes have to be associated with the rest of the EC subset components (Boyatzis, 1982). An SLP working with PTs can be used to inspire an intrinsic motivation toward improvement in ECs. These general results are aligned with previous research that related more training with higher levels of emotional skills in students' performance (Lechner et al., 2019).

In a more detailed explanation of the results, it can be noted that the trait that predicts how negative emotions are experienced is neuroticism, although some authors argue the equal explanatory power of extraversion (Zelenski et al., 2013; Spark and O'Connor, 2020). EC is negatively related to neuroticism (Nozaki and Koyasu, 2016) because it is characterized by fostering a wide range of negative emotions, including irritability and nervous tension (McCrae and John, 1992). The SPL impacts on this trait provoke changes in the students at the neurotic pole, and because of that, it can be foreseen as a positive consequence, explained by neuroticism. These neurotic traits, however, are not fully compatible with displaying and exercising self-leadership from the resonant styles model (Goleman et al., 2004). The ECD for those identified as neurotic students implies the possibility of a conscious correction of these experiences of negative emotions and progress toward a better balance through an orientation to positive affective anticipation (Spark and O'Connor, 2020).

For many authors, extraversion is the most important explanatory factor both in the appearance and in the effectiveness of leadership (Judge et al., 2002; Spark, 2020). The fact of obtaining impacts in ECD for introverted students, precisely the group that needs it the most, is because the behavior that would emanate from their PT, without the mediation of the ECD, would be being reserved and calm in social contexts (Costa and McCrae, 1992). Progress in ECD can bring introverts closer to the extroversion benefits based on a higher emotional awareness and more adjusted behaviors within the framework of social interaction. In that sense, extraversion correlates positively with the quality of interpersonal relationships of university students (Lopes et al., 2004). Previous investigations concluded that extroverted people have a greater predisposition toward positive emotional experiences (McCrae and John, 1992; Lucas and Fujita, 2000; Fleeson et al., 2002; Zelenski et al., 2013). The SLP does not embrace the extraverted ideal (Cain, 2013), but if each student is accompanied in an appropriate reflection, then introverts can freely decide when to be introverted, and when to be extraverted socially, by strengthening their self-esteem, being clearer about their motives, and being able to practice extroverted behaviors within the framework of psychological safety and the empowerment associated with this SLP training.

The antagonistic students who participated in the SLP can similarly develop the facets associated with agreeableness, which are trust, frankness, altruism, complacency, modesty, and tenderness (Costa and McCrae, 1992). In this sense, the SLP can enhance equally the ECD in this subgroup. Trust is the starting point to generating a framework of empathy, which is indispensable for the harmonic collaboration between people that is necessary for a resonant leadership (Goleman, 2017). These results reveal that antagonistic students are aware of the consequences of provoking negative emotions around them and how essential it is to generate positive emotions in their relationships. This awareness leads them to more effective emotional management and self-leadership.

Concerning consciousness, specific improvement in the ECD of students with lack of direction can be understood as an impact in terms of a more adjusted knowledge of reality and its operational norms, which displays higher levels of emotional self-control (Muthu et al., 2010), where self-control is one out of five essential components of leadership (Goleman, 2017). Previous studies found that the significance of educational experience influences engagement (Perkins et al., 2010), academic performance (Trapmann et al., 2007), as well as achievements (Goldberg, 2001). Moreover, this impact can be considered very relevant for graduate students because consciousness is also the factor that has more influence on professional success (Barrick et al., 2001).

No significant differences in the ECD of students with the closed to experience trait were identified. Previous research considered this PT as related to lower academic performance in general (De Raad and Schouwenburg, 1996). On the other hand, openness to experience is related to inferring values from reality, generating new ideas and projects, and in general, promoting curiosity about new things. Therefore, the SLP and the associated *self-intentional change* (Boyatzis and Akrivou, 2006), which, according to this program design, has to be specified as part of ECD, is not more effective in inspiring students with the closed to experience trait, making them rethink their relationship with external reality. The consequences of not obtaining ECD in those students can be interpreted as them not being equally competent to respond with willingness and know-how to take advantage of the windows of opportunity that appear in life, taking advantage when the opportunity appears (Kotter, 2012). Therefore, a positive open attitude would be the most appropriate approach. This is repeatedly made explicit in the workshops of the program: that it is very important to have a constructive attitude toward life and answer *yes*, and that EC is related to openness to experience (Nozaki and Koyasu, 2016).

Finally, expanding the EC by improving the ECD in graduate students whose PTs are far from the ideal in the professional profile can revert to a better self-leadership approach for them and in a more positive resonance (Goleman et al., 2004) in university training, where the quality of interpersonal relationships is very important (Lopes et al., 2004), as well as the enjoyment of social situations, as it is for anyone else (Watson and Clark, 1997).

## Limitations

The collection of data from self-reported sources presents a possible internal bias component, even for the protagonist participants themselves, along with the possibility of being conditioned by their attitudes and motivations (Bar-On, 1997 cited in Alburquerque et al., 2018).

Secondly, it is necessary to complement these findings by working with larger samples and with completely experimental approaches. Since SLP was a voluntary program, the target population was very large, and so it was impossible to know in anticipation which students will be enrolled. It was not possible to prepare initial interviews with all students, in performance tests or audio-visual records, that would allow observing the ECs or how the PTs are manifested, which would allow a more in-depth analysis of the total population studied.

The analysis of the differentiated effects of an SLP depending on PTs does not take into account the possibility of interaction between personality traits.

## Conclusions

The incorporation of an SLP in graduate education helps participating students to know their five PTs and to identify who can be more benefited by participating in it. Additionally, an SLP can be an easy solution to be implemented, which can complement the conventional curricula and, in this way, help in the development by mixing conventional technical skills with ECs. An SLP, from a student-centered approach, that considers personal characteristics through PTs helps in promoting student-centered training, adopting a more comprehensive perspective on graduate education.

Beyond the service that the knowledge of the PTs themselves offers to graduate students in terms of self-knowledge, there is the contribution of this self-knowledge in self-leadership by competencies (Montalvo, 2020). When students participate in an SLP, knowledge of their EC and PTs can be beneficial in the contexts of regular courses beyond the SLP. Despite the approach of learning by competences promoted by the European Higher Education Area, the method that predominates today in graduate education is still the transmission of knowledge (Saavedra and Opfer, 2012). Therefore, from the point of view of teachers who design formative experiences for students, part of their work consists of understanding what characteristics they have and how the students learn (Estrada et al., 2019). Moreover, it is necessary to have more precise information of how class groups are constituted and what they need based on their PTs. In that sense, and in a complementary way, a deeper understanding of how emotions are activated in the teaching–learning processes is required, and also a greater understanding of the variables that are determinant in the degree of involvement and activation of students (Pekrun and Perry, 2014).

Going down to the reality of the university classrooms, not only is the coherence between the final graduate profile and the more adjusted related PTs often not considered, but there is also evidence that only regular class attendance does not imply by itself the development of transversal or generic competences (Sánchez Elvira et al., 2010; Gallifa, 2011; Pérez et al., 2013; Michavila et al., 2016). Positive emotions are fundamental in the quality of educational experiences (Pekrun and Perry, 2014; citing Knoop, 2011; Oriol et al., 2016) and are the way, from the freedom of each individual, of acquiring all competences, both technical and emotional, that make possible having the prosocial behaviors that university graduate students need in order to develop integrally in all facets of life and throughout their lifetimes.

## Data Availability Statement

The raw data supporting the conclusions of this article will be made available by the authors, without undue reservation.

## Ethics Statement

The studies involving human participants were reviewed and approved by this article has been reviewed and approved; report 19200004D by the Research Ethics Committee (CER), an organ of the Faculty of Health Sciences at Blanquerna, Ramon Llull University. The patients/participants provided their written informed consent to participate in this study.

## Author Contributions

All authors listed have made a substantial, direct and intellectual contribution to the work, and approved it for publication.

## Conflict of Interest

The authors declare that the research was conducted in the absence of any commercial or financial relationships that could be construed as a potential conflict of interest.
